# Frequent variations in cancer-related genes may play prognostic role in treatment of patients with chronic myeloid leukemia

**DOI:** 10.1186/s12863-015-0308-7

**Published:** 2016-01-27

**Authors:** Alexander V. Lavrov, Ekaterina Y. Chelysheva, Svetlana A. Smirnikhina, Oleg A. Shukhov, Anna G. Turkina, Elmira P. Adilgereeva, Sergey I. Kutsev

**Affiliations:** Laboratory of Mutagenesis, Federal State Budgetary Institution “Research Centre for Medical Genetics”, Moskvorechie, 1, Moscow, 115478 Russia; Department of Molecular and Cellular Genetics, State Budgetary Educational Institution of Higher Professional Education “Russian National Research Medical University named after N.I. Pirogov” of Ministry of Health of the Russian Federation, Moscow, Russia; Scientific and Advisory Department of Chemotherapy of Myeloproliferative Disorders, Federal State-Funded Institution National Research Center for Hematology of the Ministry of Healthcare of the Russian Federation, Moscow, Russia

**Keywords:** Genetic variation, Chronic myeloid leukemia, TKI resistance, Whole exome sequencing, Biomarkers, Prognostic factors

## Abstract

**Background:**

Genome variability of host genome and cancer cells play critical role in diversity of response to existing therapies and overall success in treating oncological diseases. In chronic myeloid leukemia targeted therapy with tyrosine kinase inhibitors demonstrates high efficacy in most of the patients. However about 15 % of patients demonstrate primary resistance to standard therapy. Whole exome sequencing is a good tool for unbiased search of genetic variations important for prognosis of survival and therapy efficacy in many cancers. We apply this approach to CML patients with optimal response and failure of tyrosine kinase therapy.

**Results:**

We analyzed exome variations between optimal responders and failures and found 7 variants in cancer-related genes with different genotypes in two groups of patients. Five of them were found in optimal responders: rs11579366, rs1990236, rs176037, rs10653661, rs3803264 and two in failures: rs3099950, rs9471966. These variants were found in genes associated with cancers (*ANKRD35*, *DNAH9*, *MAGEC1*, *TOX3*) or participating in cancer-related signaling pathways (*THSD1*, *MORN2*, *PTCRA*).

**Conclusion:**

We found gene variants which may become early predictors of the therapy outcome and allow development of new early prognostic tests for estimation of therapy efficacy in CML patients. Normal genetic variation may influence therapy efficacy during targeted treatment of cancers.

**Electronic supplementary material:**

The online version of this article (doi:10.1186/s12863-015-0308-7) contains supplementary material, which is available to authorized users.

## Background

Chronic myeloid leukemia (CML) accounts for about 10 % of all leukemias. The primary mutation in blood stem cell is induced by the translocation between chromosomes 9 and 22 leading to the formation of the so-called Philadelphia chromosome (Ph+) – an aberrant chromosome 22. As a result of this translocation two genes are fused: *ABL* (chromosome 9) and *BCR* (chromosome 22) [1]. *BCR*/*ABL* gene produces atypical tyrosine kinase – BCR/ABL which activates MAPK pathway and induces cell proliferation, blocks apoptosis and leads to genome instability resulting in further development of the disease.

Till late 1990s the prognosis for the patients with CML was poor. The disease progressed gradually to the accelerated phase, blast crisis and finally death. Invention of BCR/ABL tyrosine kinase inhibitor (TKI) made the disease well controlled and dramatically improved patient’s survival. The first TKI – imatinib – has become the standard therapy of CML since its FDA approval in 2001. Patient’s survival considerably improved from 7.5 years after diagnosis before imatinib invention to 17.5 years now-a-days [2]. However some patients still remain resistant to TKI therapy. The rate of primary resistance is in the range of 5–20 % [3, 4]. According to ELN recommendations 2013 [5] CML patients on TKI therapy are assigned to optimal responders, failures and “warning” group. Optimal responders may continue the same treatment, failures need treatment changes, “warning” group needs careful observation with the readiness to change treatment options. Currently available prognostic scores – Sokal and EUTOS may help to predict patients with worse clinical outcome on TKIs although some studies failed to demonstrate predictive value of EUTOS score for different clinical outcomes including long-term overall survival rate [6, 7]. Therefore additional clinically relevant prognostic markers for CML patients are extremely important. High genetic variability of cancer cells in CML is difficult for analysis and selection of prognostic markers.

In this work we perform whole exome sequencing (WES) of newly diagnosed CML patients and analyze exomic variations which can serve as early predictive markers of the high and low efficacy of TKI therapy.

## Results

### Characteristics of the patients

Twenty three patients were available with follow-up data at 6 months after beginning TKI treatment (11 males and 12 females; mean age – 42.4 years). Two patients (8.7 %) were classified as failures, 7 patients (30.4 %) were assigned to warning group and other 14 patients demonstrated optimal response according to ELN recommendations 2013. Blood samples from 8 patients were taken for further analysis: 4 optimal responders and 4 patients with TKI inefficacy (2 failures and 2 patients from the warning group). Frontline therapy was imatinib in 3 optimal responders and 3 non responders, and nilotinib in 1 responder and 1 non responder. Two failures were switched to nilotinib and dasatinib after 2.5 and 4 months of imatinib therapy. The detailed description of the patients included in the study is available in the Additional file 1: Table S1. Blood samples were drawn before starting TKI therapy when vast majority of nucleated blood cells were leukemic. Paired blood samples were drawn from 3 optimal responders after 6 months of TKI therapy during relapse for analysis of normal exomes. All 11 blood samples were sequenced.

### Whole exome sequencing

In total about 55 millions of nucleotides in targeted sequences were covered in average 20×. Sequences were aligned to hg19. Ion Torrent Variant Caller plugin was used to call variants. In each of 11 samples we found from 33,657 to 38,636 homo- and heterozygous variants (SNVs), including single nucleotide variants and insertions and deletions. All the variations are available at European Nucleotide Archive (http://www.ebi.ac.uk/ena/data/view/PRJEB9857). There were about 5300 unique variants in each sample. All 116,508 variants were merged in one table for further annotation and analysis. Variants were found in 17,741 genes. In each gene we found from at least one variant in one or more samples up to 345 variants (*MUC16*). The full list of genes with number of found variants is available in Additional file 2: Table S2. Pathway enrichment analysis using Gorilla (ver. Mar 28, 2015) [8] revealed possibly damaged by SNVs cellular pathways: antigen processing and presentation of endogenous peptide antigen via MHC class I via ER pathway, TAP-independent (*p* = 1.8 × 10^−7^), interferon-gamma-mediated signaling pathway (*p* = 9.6 × 10^−8^) and O-glycan processing (*p* = 1.9 × 10^−13^); functions: peptide antigen binding (*p* = 1.8 × 10^−10^), MHC class II receptor activity (*p* = 2.1 × 10^−7^); and structures: MHC protein complex (*p* = 3.7 × 10^−11^), integral component of lumenal side of endoplasmic reticulum membrane (*p* = 5.2 × 10^−11^), Golgi lumen (*p* = 5 × 10^−13^) and ER to Golgi transport vesicle membrane (*p* = 7.7 × 10^−10^). The full list of the enrichment analysis results is available in Additional file 1: Table S3.

### Variant analysis

We were looking for variants which were present in all samples of one group and absent in all samples from the opposite group. We performed such a filtering for optimal responders and for the failures separately. In leukemic cells of all four optimal responder we found 11 333 variants in total. These variants included both homozygous and heterozygous SNPs. 152 of them were not present in either of leukemic samples from patients with failure of the response. We further filtered missense and splice-site variants and also variants in regulatory regions and we got only 27 variants. By manual verification of sequenced reads in IGV browser [9, 10] we confirmed 21 variant. Based on GeneCards descriptions (available in October, 2014) we selected 11 variants in genes known to be associated with cancers (*ANKRD35* – rs11579366, *ATP7B* – rs732774, COSM147691, *IGHV4*-*31* – rs61995642, *ANPEP* – rs25651, *DNAH9* – rs1990236, *CCDC165* – rs632423, *MAGEC1* – rs176037) or cancer-associated pathways like MAPK (*FCRL3* – rs7522061, *LRP2* – rs2229263, *KLB* – rs4975017) or cellular processes frequently deteriorated in cancer like DNA integrity control (*FANCD2* – rs3864017). We selected 3 variants (***ANKRD35*** – rs11579366, ***DNAH9*** – rs1990236, ***MAGEC1*** – rs176037) positive for either SIFT [11] or PolyPhen [12] score to validate them with Sanger sequencing.

We also selected additional variant previously not described in dbSNP135 which we confirmed manually with IGV browser. It was in breast cancer associated ***TOX3*** (rs10653661 according to dbSNP141). This variant was also selected for confirmation by Sanger sequencing.

Another filtering option was to select only homozygotes in all 4 optimal responders. Two out of 10 of them were confirmed by manual verification. One of them was annotated in the gene involved in angiogenesis, apoptosis, activation of TGF-beta (***THSD1*** – rs3803264), and was also selected.

We applied exactly the same filtering pipe-line to the set of data from leukemic cells in the group of failures. We found 8 969 variants in all four failures. Only 51 of them were not present in optimal responders. Filtering missense and spice-site variants left 15 variants. We verified manually 7 variants. GeneCards annotations did not allow to perform any specific filtering based on the gene functions – some genes seem to play important role in RNA metabolism (*DHX37* – rs11558556), cell growth and proliferation (*ADRA1A* – rs1048101, CM064954) or immune system (*PTCRA* – rs9471966) and little is known about others (*KIAA0226L* – rs1408184, *CCDC11* – rs35193847, *MORN2* – rs3099950, *TMX4* – rs2294248). Two variants (***MORN2*** – rs3099950, ***PTCRA*** – rs9471966) with deleterious/damaging effect according to SIFT or PolyPhen were selected for further Sanger sequencing.

Scheme of all filtering steps is available in Additional file 1: Figure S1.

### Validation of the findings with Sanger sequencing

After filtering and annotation we selected 7 variants for Sanger sequencing. Five of them (rs11579366, rs1990236, rs176037, rs10653661, rs3803264) were typical for optimal responders, two (rs3099950, rs9471966) – for failures. Sanger sequencing confirmed WES findings except one discordant result. Sanger sequencing detected heterozygote for rs10653661 in *TOX3* in one failure previously reported by WES as homozygous reference. Detailed analysis of the WES reads revealed low coverage of this particular locus and allelic variant (insertion of TTTCT) was present in the only read. Obviously it was not reported by automatic variant calling and it was not also considered during manual verification with IGV. The only read with mutation is always a very unreliable finding to be considered as a real variant.

## Discussion

Targeted tyrosine-kinase inhibitors dramatically changed efficacy of CML treatment and overall survival rates. However there are about 20 % of CML patients failing to achieve optimal response to TKI therapy. In this work we applied WES in order to find genomic variants which could explain differences in TKI inefficacy. WES is very popular in studying cancers including myeloid neoplasia, however there are no published studies about the differences between groups of CML patients with optimal and non-optimal response to standard TKI therapy [13]. This approach has the benefits of the unbiased search of the independent prognostic markers. CML patients receiving TKI therapy were assigned to either optimal responders or non-optimal/failures according to ELN criteria 2013. WES was performed in 8 patients and paired recovered samples. Bioinformatic analysis revealed classic long-tail distribution of the genes according to the number of variants found in them. Annotation of the top-100 mutated genes (from 41 to 345 mutations per gene) revealed that these genes are important for cellular processes and functions connected with the activity of the immune system. Knowledge about involvement of specific pathways allows developing gene-expression panels for signaling pathway profiling which may serve as an independent molecular marker [14].

However our aim was to find direct single-gene variations which can explain and predict response to the therapy. Multiple approaches can be applied to prioritize possible targets among thousands of SNVs found in individual exomes. We focused on the possible prognostic effect and expected to find variants with strong association with one of the treatment outcomes. We filtered exonic variants (27 in optimal responders and 15 in failures) which were either missense or otherwise potentially involved in gene functioning. We selected either missense variants with predicted damaging effect on protein function, or upstream/downstream regulatory variants. These manually confirmed variants in cancer-associated genes were selected for further Sanger resequencing. Below we describe all genes with found variants.

*ANKRD35* (Ankyrin Repeat Domain-Containing Protein 35) is a protein coding gene. Genecards annotated in October 2014 its association with chronic lymphocytic leukemia. This gene is expressed in normal human tissues, including bone marrow, whole blood and white blood cells but nothing is known about its function and role in cancer pathogenesis of its variants [15]. We found *g.1:145562293G*>*C* substitution (rs11579366) in responders but not in failures. This variant has different frequencies of alleles in different populations. According to “The 1000 Genomes Project (Phase 3)” the allele frequencies are *G* = 0.2912 in GBR but *G* = 0.8802 in ACB (data obtained on April 28, 2015). It is a promising prognostic factor because with that high population frequency we though found opposite homozygotes in failures and optimal responders. This *G*>*C* substitution has “probably damaging” PolyPhen score (0.993).

*DNAH9* (Dynein, Axonemal, Heavy Chain 9) encodes the heavy chain subunit of axonemal dynein, a large multi-subunit molecular motor. *DNAH9* interacts with B-cell CLL/lymphoma 6 (BCL6). BCL6 keeps B-cells in a proliferative state in the germinal center, and down-regulation of BCL6 is required for B-cell maturation and differentiation. The dysregulation of BCL6 is implicated in lymphomagenesis [16]. A total of 28 records are available in ClinVar with different variants of *DNAH9* found in melanoma and lung cancer (April 28, 2015). Heterozygous mutation in splicing site of *DNAH9* (*NM*_*001372.3:c.10242*+*5G*>*A*) was reported in one of the cases with atypical CML [17]. We found a missense variant (rs1990236) *g.17:11865462G*>*A*. This variant has a highly deleterious SIFT score (0.02). Its population frequency is from as low as 0.07 in YRI up to 0.3 in PEL being 0.2 in European populations.

*MAGEC1* (Melanoma Antigen Family C, 1) is a protein coding gene associated with myeloma [18], digestive tract carcinomas [19], glioblastoma [20] and melanoma [21]. It is a member of the melanoma antigen gene (MAGE) family. The proteins of this family are tumor-specific antigens that can be recognized by autologous cytolytic T-lymphocytes [22]. MAGEC1 helps cancer cells in multiple myeloma to escape from immune-surveillance, and its expression stimulates proliferation of cancer cells [23]. The variant we found (rs176037) is the *g.X:140993642 C*>*T* substitution and it has a deleterious SIFT score of 0.01. T allele frequency is about 0.5 in European populations.

We also chose variant not previously described in databases even if they did not influence the coding sequence directly. *TOX3* (High Mobility Group Box Family Member 3) is the protein coding gene. It contains a HMG-box, and may bend and unwind DNA and modify chromatin structure. Its high expression is associated with poor prognosis in breast cancer [24]. Its variant rs3803662 is associated with breast cancer [25] and multiple primary cancers [26]. TOX3 protects against cell death by inducing antiapoptotic and repressing pro-apoptotic transcripts. It stimulates transcription from the estrogen-responsive or BCL-2 promoters. Among optimal responders we found one homozygote and other heterozygotes for rs10653661 – insertion of 5 nucleotides in 3′-UTR *g.16:52472421*_*2insTTTCT*. This insertion was not described in dbSNP135 which we used for annotating variants before their filtration. And we chose this variant as a new, not previously described finding. This variant got its accession in dbSNP141 and population frequencies are now available for it: from 0.24 in CDX to 0.85 in YRI with 0.42–0.44 in Europeans. The gene plays an important role in cellular processes involved in cancer and its variants are associated with different cancers and diseases.

We also filtered homozygotes present in all optimal responders while all failures were wild-type homozygotes. One of the variants was annotated as upstream gene variant in *THSD1* (Thrombospondin, Type I, Domain Containing 1). It is a surface marker of hematopoietic progenitors and endothelial cells [27]. *THSD1* is methylated and down regulated in colorectal cancer [28] and esophageal squamous cell carcinoma. In all optimal responders we found rs3803264 in a homozygous form (AA) while all failures were wild-type homozygotes (GG). This *g.13:52971893G*>*A* substitution is synonymous in most of the *THSD1* transcripts (NM_018676, NM_199263), but NM_018676 can generate a shorter protein (uc010tgz.2 length = 473 instead of uc001vgo.3 length = 852) in which case the variant is considered as upstream gene variant. It has a high population frequency of 0.65 in Europeans.

All the above described variants were present in the same genotype in paired samples of optimal responders before the beginning and after 6 months of TKI therapy. This is why we consider these variants to be present in the constitutive genome but not leukemic-specific somatic mutations.

In failures we found only two variants following the same criteria as described above for the optimal responders.

*MORN2* (Membrane Occupation and Recognition Nexus Repeat Containing 2) is a protein coding gene. There are five MORN-repeat containing genes in humans. MORN motifs are necessary for binding junctophilins to plasma membrane and endoplasmic reticulum [29]. Human MORN2 facilitates phagocytosis-mediated restriction of Mycobacterium tuberculosis in macrophages and is important for LC3-associated phagocytosis [30]. MORN4 interacts with class III myosins and may be transported to filopodia of moving cells [31]. We found rs3099950 in heterozygous form in all failures but in none of the responders. This missense substitution *g.2:39109558G*>*A* has been registered with different frequencies in populations from 0.0 in many to 0.18 in PJL and has the frequency of ~0.14 in Europeans.

*PTCRA* (Pre T-Cell Antigen Receptor Alpha) encodes a protein that is found in immature T-cells. PTCRA is the member of the pre-T-cell receptor complex, which regulates early T-cell development. This gene along with many others undergoes subsequent demethylation, remethylation and repression in appropriate stages of differentiation. PTCRA is required for T-cell receptor rearrangement and after this stage of T-cell development it needs to be silenced in mature CD4+ cells to avoid MHC class I binding [32]. PTCRA is also a member of NOTCH1 signaling pathway which is an important regulator of T-cell differentiation [33]. It was shown that *PTCRA* is expressed in acute promyelocytic leukemia significantly [34]. All failures in our study were heterozygotes for rs9471966 – *g.6:42891022G*>*A* substitution. It has both SIFT and PolyPhen deleterious scores. Allele A has the lowest frequency in CHS – 0.01 and the highest in ESN 0.44, and with ~0.25 in Europeans. We consider it to be a good target for both prognostic purposes and explanation of therapy failure.

All seven variants have high population frequency in at least some populations. Since we are not looking for the variants associated with CML but rather with its sensitivity to TKI treatment, high population frequency of the potential markers and dramatic difference in their frequency between optimal responders and failures is very promising for finding a highly sensitive and specific prognostic marker. We suggest that TKI therapy may be more successful for patients carrying the following markers all together or in some combination: rs11579366 (C/C or C/G), rs1990236 (G/A), rs176037 (T/T or C/T), rs10653661 (A/ATTTCT or ATTTCT/ATTTCT), rs3803264 (A/A) and reference variants for rs3099950 (G/G) and rs9471966 (G/G). However real prognostic value of these markers and their possible combinations can be clarified only in further prospective studies.

## Conclusions

In conclusion we performed whole exome sequencing of 8 CML patients. Four of them demonstrated optimal response to standard TKI therapy and another 4 were considered as failures. We filtered out from whole exome data 7 variants (6 SNVs and one 5 nucleotide insertion) which reflect heterogeneity of treatment efficacy in patients with CML and may help to select patients with primary resistance to TKI therapy. As far as genes with the selected variants associate with other types of cancers and found variants have predicted damaging effect their possible functional effect should be carefully studied. To further validate functional role and prognostic value of the variants it is necessary to perform retrospective and prospective studies in groups of CML patients.

## Methods

Newly diagnosed patients with Ph+ CML in chronic phase at diagnosis have been included in this study at Federal State-Funded Institution National Research Center for Hematology of the Ministry of Healthcare of the Russian Federation (Moscow, Russia) starting from June, 2012. Peripheral blood samples were collected at diagnosis after signing informed consent “Exome and transcriptome analysis of leukemic cells in chronic myeloid leukemia”. The study was conducted according to the provisions of the Declaration of Helsinki. All patients received standard treatment with TKIs and were examined at regular time periods – 3, 6, 9 and 12 months during the 1-st year of observation. The examination included clinical evaluation, complete blood count, cytogenetic and molecular tests which measured *BCR*-*ABL* expression by quantitative PCR. Patients were assigned to the groups of either optimal responders or failures according to ELN recommendations 2013 [5]. For the purpose of our study we evaluated response at 6 months. All failures were checked for the absence of mutations in the ABL-kinase domain of the *BCR*-*ABL* by sequencing. DNA extracted from blood samples was used for WES. WES was performed using Ion Torrent Personal Genome Machine (Thermo Fisher Scientific, Waltham, MA, USA) with 200 bp reads. Exome enrichment was performed using Ion TargetSeq™ Exome Kit which captures about 30,000 protein-coding and RNA genes and predicted microRNA-binding sites. Sequencing analysis was done in Torrent Suite software v.3.6.2. Single nucleotide variants (SNVs) were annotated with ANNOVAR [35], Partek Genomics Suite (Partek, St. Louis, Missouri, USA) and Variant Effect Predictor tool [36]. Variants identified by whole exome sequencing, were validated by Sanger sequencing. Methods in more details are described in Additional file 1.

### Availability of supporting data

The data set supporting the results of this article is available in the European Nucleotide Archive repository, # PRJEB9857 http://www.ebi.ac.uk.

The data sets supporting the results of this article are included within the article and its additional files: Additional files 1 and 2 which are referenced the main text.

## Additional files

Additional file 1:
**Supplementary Methods. Table S1.** Characteristics of patients. **Table S3.** Pathway enrichment analysis. **Figure S1.** Filtering variants. (DOC 268 kb) Additional file 2: Table S2. List of Genes and number of variants in them. (CSV 1370 kb)
