# Supplementary material for: Frequent variations in cancer-related genes may play prognostic role in treatment of patients with chronic myeloid leukemia
Source: BMC Genet. 2016 Jan 27;17(Suppl 1):14. doi: 10.1186/s12863-015-0308-7 (PMC4895599; doi:10.1186/s12863-015-0308-7)
Supplement: Additional file 1: — Supplementary Methods. Table S1. Characteristics of patients. Table S3. Pathway enrichment analysis. Figure S1. Filtering variants. (DOC 268 kb) [file 12863_2015_308_MOESM1_ESM.doc]

**Supplementary information**

**Frequent variations in cancer-related genes may play prognostic role in treatment of patients with chronic myeloid leukemia**

Alexander V. Lavrov, Ekaterina Y. Chelysheva, Svetlana A. Smirnikhina, Oleg A. Shukhov, Anna G. Turkina, Elmira P. Adilgereeva, Sergey I. Kutsev

**Contents of Supplementary Information**

Supplementary Methods

Table S1. Characteristics of patients.

Table S2. List of Genes and number of variants in them. File: Table S2.csv

Table S3. Pathway enrichment analysis.

Figure S1. Filtering variants.

**Supplementary Methods**

***Exome sequencing***

We extracted genomic DNA from blood samples using commercial kit DNA-Sorb-B Kit (InterLabService, Moscow, Russia, # К1-2-100). The exome libraries were generated starting from 1 ug of gDNA using Life Technologies Ion TargetSeq™ Exome Kits (#4477743, Thermo Fisher Scientific, Waltham, MA, USA) with fragment size of 200–250 bp. The libraries were sequenced using the Ion Torrent Personal Genome Machine with 200 bp reads and the Ion PGM™ 200 Sequencing Kit (#4474004).

***Whole-exome sequencing analysis***

Raw data analysis, base calling and alignment to hg19 (GRCh37 – <http://updates.iontorrent.com/reference/hg19.zip> provided by Life Technologies) were performed using the Torrent Suite (TS) software v. 3.6.2. BAM-files generated by TS were analyzed by Variant Caller plugin v3.6.63335. The .bed file for variant calling was provided together with the Ion TargetSeq™ Exome Kit.

***Pipeline for variants discovery***

The list of variants, generated by Variant Caller plugin from each analyzed sample, contained over 30000 variants, including not only SNVs but also insertion and deletions (indels) from one to several nucleotides in length. All variants were deposited in EVA (<http://www.ebi.ac.uk/eva/>) accession PRJEB9656. These variants were annotated with ANNOVAR, Partek Genomics Suite (Partek, St. Louis, Missouri, USA) and Variant Effect Predictor tool. Partek Genomics Suite and MS Office Excel were used for filtering variants. The pipeline in details is described in Figure S1 and in Results.

***Sanger sequencing***

To validate variants identified by whole-exome sequencing, two primers, upstream and

downstream the variant, were used from pre-designed library using Primer Designer™ Tool (Thermo Fisher Scientific, Waltham, MA, USA). Primer sequences and catalogue numbers are available below:

| Name | Length | Sequence | Life Technologies catalogue # |
| --- | --- | --- | --- |
| ANKRD35 F | 507 | GCAACAGTAACTTGCTGGAGGAGTT | Hs00399911_CE |
| ANKRD35 R |  | CTTCCCCAGGTCTTGCTCCAGA |  |
| DNAH9 F | 511 | ATAACTCTTGTCTGCCTGGGTGC | Hs00162006_CE |
| DNAH9 R |  | ATTCCAAGCTTTACCTGTGTGTCCCA |  |
| MAGEC1 F | 492 | TTCTCCTGAGAGCGACGACAC | Hs00488640_CE |
| MAGEC1 R |  | CGGCTCACAGGAATCTGGAGT |  |
| TOX3 F | 512 | TTCAGATGTGGTGTGCCTTTTAAAT | Hs00155834_CE |
| TOX3 R |  | GCTATCTGAACTCCTGAAGCCCTAA |  |
| THSD1 F | 508 | TGGCACCATCACCAGTTTGTATCC | Hs00132739_CE |
| THSD1 R |  | CAACCAGTCCCAGGGAACACTA |  |
| MORN2 F | 544 | AAAGCAAAGCTTGAAAGTAGAGGAT | Hs00193190_CE |
| MORN2 R |  | GGACACACAAGCACAAACTAGAAA |  |
| PTCRA F | 509 | GAACCCTAGCCTACAACACAATGCT | Hs00274873_CE |
| PTCRA R |  | TGGACACCCTTCATGCCTTTGTG |  |

Amplicons were sequenced from forward and reverse primer by Sanger sequencing and the variant was identified using Chromas 2 Software.

|  |  |  | Laboratory test at Dx | |  |  |  | Laboratory test at 6 months after TKI start | | |  |  |
| --- | --- | --- | --- | --- | --- | --- | --- | --- | --- | --- | --- | --- |
| # | Years at Dx | Group | Cytogenetics, Ph+, % | BCR-ABL^IS^, % | Frontline TKI | Change of therapy, days | 2-nd TKI | "6 mths" in days | Cytogenetics, Ph+, % | BCR-ABL^IS^, % | Dosage at the beginning, mg | Dosage corrected, mg |
| 1 | 28 | Optimal | 100 | 69 | Nilotinib | - | - | 181 | 0 | 0.01 | 600 | 600 |
| 2 | 75 | Optimal | 100 | - | Imatinib | - | - | 190 | - | 0.01 | 400 | 400 |
| 3 | 37 | Optimal | 100 | 69.9 | Imatinib | - | - | 188 | 0 | 0.58 | 400 | 400 |
| 4 | 53 | Optimal | 99 | 74.4 | Imatinib | - | - | 181 | 0 | 1.09 | 400 | 400 |
| 5 | 67 | Failure | 97 (FISH) | 48.5 | Imatinib | 74 | Nilotinib | 178 | 59 | 12.82 | 400 | 600 |
| 6 | 32 | Failure | 100 | - | Imatinib | - | - | 179 | 35 | 2.25 | 400 | 600 |
| 7 | 29 | Failure | - | 24.6 | Nilotinib | - | - | 197 | 17 | 3.81 | 800 | - |
| 8 | 62 | Failure | 100 | 40.2 | Imatinib | 129 | Dasatinib | 217 | 10 | 2.26 | 400 | 100 |

Table S1. Characteristics of patients.

Dx – diagnosis; Ph+,% - percentage of metaphases with Ph-chromosome in the samples of bone marrow; BCR-ABL^IS^ – percentage of BCR/ABL expression in international score; TKI – tyrosine kinase inhibitor.

Table S2. List of Genes and number of variants in them. File: Table S2.csv

| **GO term** | **Process description** | **[P-value](http://cbl-gorilla.cs.technion.ac.il/GOrilla/0luhpyj5/GOResultsPROCESS.html#p_value_info)** | **[FDR q-value](http://cbl-gorilla.cs.technion.ac.il/GOrilla/0luhpyj5/GOResultsPROCESS.html#fdr_info)** | **[Genes](http://cbl-gorilla.cs.technion.ac.il/GOrilla/0luhpyj5/GOResultsPROCESS.html#genes_info)** |
| --- | --- | --- | --- | --- |
| [GO:0016266](http://www.godatabase.org/cgi-bin/amigo/go.cgi?query=GO:0016266&view=details) | O-glycan processing | 1.99E-13 | 2.57E-9 | MUC12, MUC5B, MUC2, MUC3A MUC4, MUC21, MUC17, MUC19, MUC6, MUC16 |
| [GO:0006493](http://www.godatabase.org/cgi-bin/amigo/go.cgi?query=GO:0006493&view=details) | protein O-linked glycosylation | 5.14E-11 | 3.33E-7 | MUC12, MUC5B, MUC2, MUC3A MUC4, MUC21, MUC17, MUC19, MUC6, MUC16 |
| [GO:0060333](http://www.godatabase.org/cgi-bin/amigo/go.cgi?query=GO:0060333&view=details) | interferon-gamma-mediated signaling pathway | 9.63E-8 | 4.16E-4 | HLA-A, HLA-DRB1, HLA-C, HLA-B, HLA-DQB1, HLA-DQA1, HLA-DPA1 |
| [GO:0002486](http://www.godatabase.org/cgi-bin/amigo/go.cgi?query=GO:0002486&view=details) | antigen processing and presentation of endogenous peptide antigen via MHC class I via ER pathway, TAP-independent | 1.83E-7 | 5.94E-4 | HLA-A, HLA-C, HLA-B |
| [GO:0043687](http://www.godatabase.org/cgi-bin/amigo/go.cgi?query=GO:0043687&view=details) | post-translational protein modification | 6.66E-7 | 1.73E-3 | MUC12, MUC5B, MUC2, MUC3A MUC4, MUC21, MUC17, MUC19, MUC6, MUC16 |
| [GO:0006486](http://www.godatabase.org/cgi-bin/amigo/go.cgi?query=GO:0006486&view=details) | protein glycosylation | 1.61E-6 | 3.47E-3 | MUC12, MUC5B, MUC2, MUC3A MUC4, MUC21, MUC17, MUC19, MUC6, MUC16 |
| [GO:0043413](http://www.godatabase.org/cgi-bin/amigo/go.cgi?query=GO:0043413&view=details) | macromolecule glycosylation | 1.61E-6 | 2.98E-3 | MUC12, MUC5B, MUC2, MUC3A MUC4, MUC21, MUC17, MUC19, MUC6, MUC16 |
| [GO:0002484](http://www.godatabase.org/cgi-bin/amigo/go.cgi?query=GO:0002484&view=details) | antigen processing and presentation of endogenous peptide antigen via MHC class I via ER pathway | 1.82E-6 | 2.95E-3 | HLA-A, HLA-C, HLA-B |
| [GO:0070085](http://www.godatabase.org/cgi-bin/amigo/go.cgi?query=GO:0070085&view=details) | glycosylation | 2.06E-6 | 2.97E-3 | MUC12, MUC5B, MUC2, MUC3A MUC4, MUC21, MUC17, MUC19, MUC6, MUC16 |
| [GO:0002480](http://www.godatabase.org/cgi-bin/amigo/go.cgi?query=GO:0002480&view=details) | antigen processing and presentation of exogenous peptide antigen via MHC class I, TAP-independent | 6.31E-6 | 8.18E-3 | HLA-A, HLA-C, HLA-B |
| [GO:0019885](http://www.godatabase.org/cgi-bin/amigo/go.cgi?query=GO:0019885&view=details) | antigen processing and presentation of endogenous peptide antigen via MHC class I | 1.01E-5 | 1.19E-2 | HLA-A, HLA-C, HLA-B |
| [GO:0002483](http://www.godatabase.org/cgi-bin/amigo/go.cgi?query=GO:0002483&view=details) | antigen processing and presentation of endogenous peptide antigen | 1.5E-5 | 1.62E-2 | HLA-A, HLA-C, HLA-B |
| [GO:0019883](http://www.godatabase.org/cgi-bin/amigo/go.cgi?query=GO:0019883&view=details) | antigen processing and presentation of endogenous antigen | 2.14E-5 | 2.13E-2 | HLA-A, HLA-C, HLA-B |
| [GO:0002478](http://www.godatabase.org/cgi-bin/amigo/go.cgi?query=GO:0002478&view=details) | antigen processing and presentation of exogenous peptide antigen | 2.68E-5 | 2.48E-2 | HLA-A, HLA-DRB1, HLA-C, HLA-B, HLA-DQB1, HLA-DQA1, HLA-DPA1 |
| [GO:0016045](http://www.godatabase.org/cgi-bin/amigo/go.cgi?query=GO:0016045&view=details) | detection of bacterium | 2.93E-5 | 2.53E-2 | HLA-A, HLA-DRB1, HLA-B |
| [GO:0019884](http://www.godatabase.org/cgi-bin/amigo/go.cgi?query=GO:0019884&view=details) | antigen processing and presentation of exogenous antigen | 3.44E-5 | 2.79E-2 | HLA-A, HLA-DRB1, HLA-C, HLA-B, HLA-DQB1, HLA-DQA1, HLA-DPA1 |
| [GO:0030277](http://www.godatabase.org/cgi-bin/amigo/go.cgi?query=GO:0030277&view=details) | maintenance of gastrointestinal epithelium | 3.89E-5 | 2.96E-2 | MUC2, MUC4, MUC6 |
| [GO:0098543](http://www.godatabase.org/cgi-bin/amigo/go.cgi?query=GO:0098543&view=details) | detection of other organism | 3.89E-5 | 2.8E-2 | HLA-DRB1, HLA-A, HLA-B |
| [GO:0048002](http://www.godatabase.org/cgi-bin/amigo/go.cgi?query=GO:0048002&view=details) | antigen processing and presentation of peptide antigen | 5.49E-5 | 3.74E-2 | HLA-A, HLA-DRB1, HLA-C, HLA-B, HLA-DQB1, HLA-DQA1, HLA-DPA1 |
| [GO:0010669](http://www.godatabase.org/cgi-bin/amigo/go.cgi?query=GO:0010669&view=details) | epithelial structure maintenance | 9.73E-5 | 6.31E-2 | MUC2, MUC4, MUC6 |
| [GO:0022409](http://www.godatabase.org/cgi-bin/amigo/go.cgi?query=GO:0022409&view=details) | positive regulation of cell-cell adhesion | 1.34E-4 | 8.27E-2 | HLA-A, HLA-DRB1, HLA-DQB1, HLA-DQA1, HLA-DPA1, ANK3, PIEZO1 |
| [GO:0019882](http://www.godatabase.org/cgi-bin/amigo/go.cgi?query=GO:0019882&view=details) | antigen processing and presentation | 1.34E-4 | 7.89E-2 | HLA-A, HLA-DRB1, HLA-C, HLA-B, HLA-DQB1, HLA-DQA1, HLA-DPA1 |
| [GO:0044723](http://www.godatabase.org/cgi-bin/amigo/go.cgi?query=GO:0044723&view=details) | single-organism carbohydrate metabolic process | 1.38E-4 | 7.79E-2 | MUC5B, MUC12, MUC2, MUC3A, MUC4, MUC21, MUC17, MUC19, UGT1A10, MUC6, MUC16 |
| [GO:0001916](http://www.godatabase.org/cgi-bin/amigo/go.cgi?query=GO:0001916&view=details) | positive regulation of T cell mediated cytotoxicity | 1.41E-4 | 7.59E-2 | HLA-A, HLA-C, HLA-B |
| [GO:0048496](http://www.godatabase.org/cgi-bin/amigo/go.cgi?query=GO:0048496&view=details) | maintenance of organ identity | 1.94E-4 | 1.01E-1 | GPR98, USH2A |
| [GO:0098581](http://www.godatabase.org/cgi-bin/amigo/go.cgi?query=GO:0098581&view=details) | detection of external biotic stimulus | 2.26E-4 | 1.13E-1 | HLA-DRB1, HLA-A, HLA-B |
| [GO:0001914](http://www.godatabase.org/cgi-bin/amigo/go.cgi?query=GO:0001914&view=details) | regulation of T cell mediated cytotoxicity | 2.99E-4 | 1.43E-1 | HLA-A, HLA-C, HLA-B |
| [GO:0042270](http://www.godatabase.org/cgi-bin/amigo/go.cgi?query=GO:0042270&view=details) | protection from natural killer cell mediated cytotoxicity | 3.23E-4 | 1.49E-1 | HLA-A, HLA-B |
| [GO:0009595](http://www.godatabase.org/cgi-bin/amigo/go.cgi?query=GO:0009595&view=details) | detection of biotic stimulus | 3.4E-4 | 1.52E-1 | HLA-DRB1, HLA-A,HLA-B |
| [GO:0022407](http://www.godatabase.org/cgi-bin/amigo/go.cgi?query=GO:0022407&view=details) | regulation of cell-cell adhesion | 3.58E-4 | 1.55E-1 | HLA-A, HLA-DRB1, MUC22, HLA-DQB1, HLA-DQA1, HLA-DPA1, ANK3, PIEZO1 |
| [GO:0034112](http://www.godatabase.org/cgi-bin/amigo/go.cgi?query=GO:0034112&view=details) | positive regulation of homotypic cell-cell adhesion | 4.11E-4 | 1.72E-1 | HLA-A, HLA-DRB1, HLA-DQB1, HLA-DQA1, HLA-DPA1, ANK3 |
| [GO:0005975](http://www.godatabase.org/cgi-bin/amigo/go.cgi?query=GO:0005975&view=details) | carbohydrate metabolic process | 4.6E-4 | 1.86E-1 | MUC5B, MUC12, MUC2, MUC3A, MUC4, MUC21, MUC17, MUC19, UGT1A10, MUC6, MUC16, HSPG2 |
| [GO:0031295](http://www.godatabase.org/cgi-bin/amigo/go.cgi?query=GO:0031295&view=details) | T cell costimulation | 4.68E-4 | 1.84E-1 | HLA-DRB1, HLA-DQB1, HLA-DQA1, HLA-DPA1 |
| [GO:0002455](http://www.godatabase.org/cgi-bin/amigo/go.cgi?query=GO:0002455&view=details) | humoral immune response mediated by circulating immunoglobulin | 4.82E-4 | 1.84E-1 | HLA-DRB1, HLA-DQB1 |
| [GO:0031294](http://www.godatabase.org/cgi-bin/amigo/go.cgi?query=GO:0031294&view=details) | lymphocyte costimulation | 4.97E-4 | 1.84E-1 | HLA-DRB1, HLA-DQB1, HLA-DQA1, HLA-DPA1 |
| [GO:0007010](http://www.godatabase.org/cgi-bin/amigo/go.cgi?query=GO:0007010&view=details) | cytoskeleton organization | 6.07E-4 | 2.19E-1 | PCNT, DST, SYNE2, TTC40, SVIL, SPTBN5, TEX14, SYNE1, ANK3, TTN, OBSCN |
| [GO:0002711](http://www.godatabase.org/cgi-bin/amigo/go.cgi?query=GO:0002711&view=details) | positive regulation of T cell mediated immunity | 6.65E-4 | 2.33E-1 | HLA-A, HLA-C, HLA-B |
| [GO:0002381](http://www.godatabase.org/cgi-bin/amigo/go.cgi?query=GO:0002381&view=details) | immunoglobulin production involved in immunoglobulin mediated immune response | 6.73E-4 | 2.3E-1 | HLA-DRB1, HLA-DQB1 |
| [GO:0001912](http://www.godatabase.org/cgi-bin/amigo/go.cgi?query=GO:0001912&view=details) | positive regulation of leukocyte mediated cytotoxicity | 7.34E-4 | 2.44E-1 | HLA-A, HLA-C, HLA-B |
| [GO:0002716](http://www.godatabase.org/cgi-bin/amigo/go.cgi?query=GO:0002716&view=details) | negative regulation of natural killer cell mediated immunity | 8.94E-4 | 2.9E-1 | HLA-A, HLA-C, HLA-B |
| [GO:0045953](http://www.godatabase.org/cgi-bin/amigo/go.cgi?query=GO:0045953&view=details) | negative regulation of natural killer cell mediated cytotoxicity | 8.94E-4 | 2.83E-1 | HLA-A, HLA-B |
| [GO:0090286](http://www.godatabase.org/cgi-bin/amigo/go.cgi?query=GO:0090286&view=details) | cytoskeletal anchoring at nuclear membrane | 8.94E-4 | 2.76E-1 | SYNE2, SYNE1 |
| [GO:0050982](http://www.godatabase.org/cgi-bin/amigo/go.cgi?query=GO:0050982&view=details) | detection of mechanical stimulus | 9.65E-4 | 2.91E-1 | GPR98, TTN, PIEZO1 |
| **GO term** | **Function description** | **[P-value](http://cbl-gorilla.cs.technion.ac.il/GOrilla/0luhpyj5/GOResultsFUNCTION.html#p_value_info)** | **[FDR q-value](http://cbl-gorilla.cs.technion.ac.il/GOrilla/0luhpyj5/GOResultsFUNCTION.html#fdr_info)** | **[Genes](http://cbl-gorilla.cs.technion.ac.il/GOrilla/0luhpyj5/GOResultsFUNCTION.html#genes_info)** |
| [GO:0042605](http://www.godatabase.org/cgi-bin/amigo/go.cgi?query=GO:0042605&view=details) | peptide antigen binding | 1.75E-10 | 6.94E-7 | HLA-A, HLA-DRB1, HLA-C, HLA-B, HLA-DQB1, HLA-DQA1, HLA-DPA1 |
| [GO:0003823](http://www.godatabase.org/cgi-bin/amigo/go.cgi?query=GO:0003823&view=details) | antigen binding | 2.63E-8 | 5.24E-5 | HLA-A, HLA-DRB1, HLA-C, HLA-B, HLA-DQB1, HLA-DQA1, HLA-DPA1, KIR2DL3 |
| [GO:0030197](http://www.godatabase.org/cgi-bin/amigo/go.cgi?query=GO:0030197&view=details) | extracellular matrix constituent, lubricant activity | 1.83E-7 | 2.43E-4 | MUC3A, MUC4, MUC17 |
| [GO:0005201](http://www.godatabase.org/cgi-bin/amigo/go.cgi?query=GO:0005201&view=details) | extracellular matrix structural constituent | 1.95E-7 | 1.94E-4 | DSPP , MUC3A, COL5A3, MUC4, MUC17, FBN3, MUC6 |
| [GO:0032395](http://www.godatabase.org/cgi-bin/amigo/go.cgi?query=GO:0032395&view=details) | MHC class II receptor activity | 2.08E-7 | 1.66E-4 | HLA-DRB1, HLA-DQB1, HLA-DQA1, HLA-DPA1 |
| [GO:0005509](http://www.godatabase.org/cgi-bin/amigo/go.cgi?query=GO:0005509&view=details) | calcium ion binding | 1.02E-6 | 6.77E-4 | PRSS3, DST, PKD1L2, FBN3, PCDHA1, PCDHGA1, TTN,  DSPP, FLG, GPR98, HRNR, MACF1, RPH3AL, FAT1,  FAT2 |
| [GO:0046977](http://www.godatabase.org/cgi-bin/amigo/go.cgi?query=GO:0046977&view=details) | TAP binding | 1.82E-6 | 1.03E-3 | HLA-A, HLA-C, HLA-B |
| [GO:0005198](http://www.godatabase.org/cgi-bin/amigo/go.cgi?query=GO:0005198&view=details) | structural molecule activity | 2.73E-5 | 1.36E-2 | FLG, DSPP, COL5A3, MUC3A, MUC4, MUC17, FBN3, MUC6, ANK3, TTN, NEB, OBSCN |
| [GO:0008092](http://www.godatabase.org/cgi-bin/amigo/go.cgi?query=GO:0008092&view=details) | cytoskeletal protein binding | 5.55E-5 | 2.45E-2 | DST, SPTBN5, SYNE1, TTN, USH2A, NEB, OBSCN, FRG1, GPR98, SYNE2, SVIL, ANK3, RPH3AL, MACF1 |
| [GO:0042277](http://www.godatabase.org/cgi-bin/amigo/go.cgi?query=GO:0042277&view=details) | peptide binding | 7.33E-5 | 2.92E-2 | HLA-A, HLA-DRB1, HLA-C, HLA-B, HLA-DQB1, HLA-DQA1, HLA-DPA1 |
| [GO:0033218](http://www.godatabase.org/cgi-bin/amigo/go.cgi?query=GO:0033218&view=details) | amide binding | 1.14E-4 | 4.12E-2 | HLA-A, HLA-DRB1, HLA-C, HLA-B, HLA-DQB1, HLA-DQA1, HLA-DPA1 |
| [GO:0003779](http://www.godatabase.org/cgi-bin/amigo/go.cgi?query=GO:0003779&view=details) | actin binding | 2.22E-4 | 7.34E-2 | FRG1, DST, SYNE2, SVIL, SPTBN5, SYNE1, MACF1, TTN, NEB |
| [GO:0051015](http://www.godatabase.org/cgi-bin/amigo/go.cgi?query=GO:0051015&view=details) | actin filament binding | 3.14E-4 | 9.62E-2 | SYNE2, SVIL, SYNE1, MACF1, TTN |
| [GO:0005518](http://www.godatabase.org/cgi-bin/amigo/go.cgi?query=GO:0005518&view=details) | collagen binding | 3.41E-4 | 9.68E-2 | DSPP, COL5A3, VWF, USH2A |
| **GO term** | **Component description** | **[P-value](http://cbl-gorilla.cs.technion.ac.il/GOrilla/0luhpyj5/GOResultsFUNCTION.html#p_value_info)** | **[FDR q-value](http://cbl-gorilla.cs.technion.ac.il/GOrilla/0luhpyj5/GOResultsFUNCTION.html#fdr_info)** | **[Genes](http://cbl-gorilla.cs.technion.ac.il/GOrilla/0luhpyj5/GOResultsFUNCTION.html#genes_info)** |
| [GO:0042605](http://www.godatabase.org/cgi-bin/amigo/go.cgi?query=GO:0042605&view=details) | peptide antigen binding | 1.75E-10 | 6.94E-7 | HLA-A, HLA-DRB1, HLA-C, HLA-B, HLA-DQB1, HLA-DQA1, HLA-DPA1 |
| [GO:0003823](http://www.godatabase.org/cgi-bin/amigo/go.cgi?query=GO:0003823&view=details) | antigen binding | 2.63E-8 | 5.24E-5 | HLA-A, HLA-DRB1, HLA-C, HLA-B, HLA-DQB1, KIR2DL3, HLA-DQA1, HLA-DPA1 |
| [GO:0030197](http://www.godatabase.org/cgi-bin/amigo/go.cgi?query=GO:0030197&view=details) | extracellular matrix constituent, lubricant activity | 1.83E-7 | 2.43E-4 | MUC3A, MUC4, MUC17 |
| [GO:0005201](http://www.godatabase.org/cgi-bin/amigo/go.cgi?query=GO:0005201&view=details) | extracellular matrix structural constituent | 1.95E-7 | 1.94E-4 | DSPP, MUC3A, COL5A3, MUC4, MUC17, FBN3, MUC6 |
| [GO:0032395](http://www.godatabase.org/cgi-bin/amigo/go.cgi?query=GO:0032395&view=details) | MHC class II receptor activity | 2.08E-7 | 1.66E-4 | HLA-DRB1, HLA-DQB1, HLA-DQA1, HLA-DPA1 |
| [GO:0005509](http://www.godatabase.org/cgi-bin/amigo/go.cgi?query=GO:0005509&view=details) | calcium ion binding | 1.02E-6 | 6.77E-4 | PRSS3, DST, PKD1L2, FBN3, PCDHA1,  PCDHGA1, TTN, DSPP, FLG, GPR98,  HRNR, MACF1, RPH3AL, FAT1, FAT2 |
| [GO:0046977](http://www.godatabase.org/cgi-bin/amigo/go.cgi?query=GO:0046977&view=details) | TAP binding | 1.82E-6 | 1.03E-3 | HLA-A, HLA-C, HLA-B |
| [GO:0005198](http://www.godatabase.org/cgi-bin/amigo/go.cgi?query=GO:0005198&view=details) | structural molecule activity | 2.73E-5 | 1.36E-2 | FLG, DSPP, COL5A3, MUC3A, MUC4, MUC17, FBN3, MUC6, ANK3, TTN, NEB, OBSCN |
| [GO:0008092](http://www.godatabase.org/cgi-bin/amigo/go.cgi?query=GO:0008092&view=details) | cytoskeletal protein binding | 5.55E-5 | 2.45E-2 | DST, SPTBN5, SYNE1, TTN, USH2A, NEB, OBSCN, FRG1, GPR98, SYNE2, SVIL, ANK3, RPH3AL, MACF1 |
| [GO:0042277](http://www.godatabase.org/cgi-bin/amigo/go.cgi?query=GO:0042277&view=details) | peptide binding | 7.33E-5 | 2.92E-2 | HLA-A, HLA-DRB1, HLA-C, HLA-B, HLA-DQB1, HLA-DQA1, HLA-DPA1 |
| [GO:0033218](http://www.godatabase.org/cgi-bin/amigo/go.cgi?query=GO:0033218&view=details) | amide binding | 1.14E-4 | 4.12E-2 | HLA-A, HLA-DRB1, HLA-C, HLA-B, HLA-DQB1, HLA-DQA1, HLA-DPA1 |
| [GO:0003779](http://www.godatabase.org/cgi-bin/amigo/go.cgi?query=GO:0003779&view=details) | actin binding | 2.22E-4 | 7.34E-2 | FRG1, DST, SYNE2, SVIL, SPTBN5, SYNE1, MACF1, TTN, NEB |
| [GO:0051015](http://www.godatabase.org/cgi-bin/amigo/go.cgi?query=GO:0051015&view=details) | actin filament binding | 3.14E-4 | 9.62E-2 | SYNE2, SVIL, SYNE1, MACF1, TTN |
| [GO:0005518](http://www.godatabase.org/cgi-bin/amigo/go.cgi?query=GO:0005518&view=details) | collagen binding | 3.41E-4 | 9.68E-2 | DSPP, COL5A3, VWF, USH2A |

Table S3. Pathway enrichment analysis.

There are 17741 genes entered. 15489 of these genes are associated with a GO term. The GOrilla database was updated on Mar 28, 2015


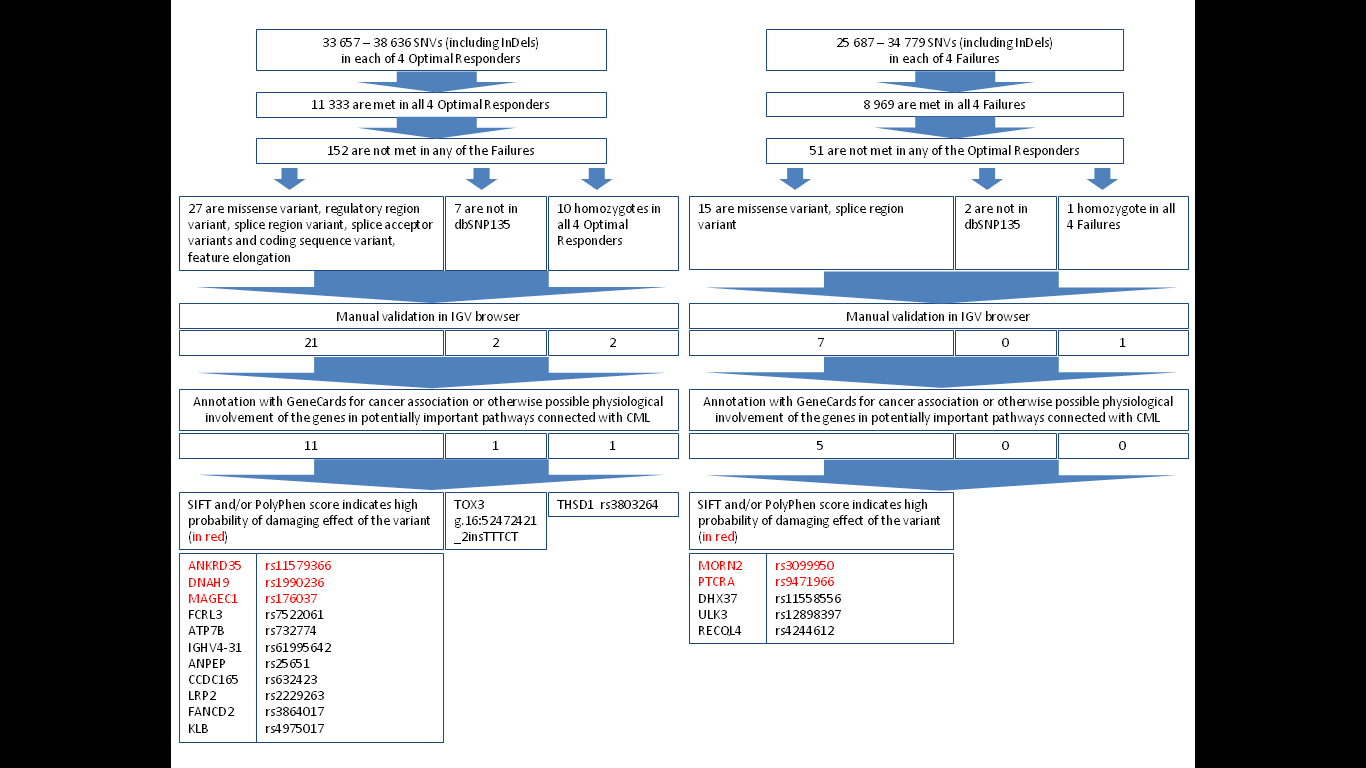


Figure S1. Filtering variants.
